# Analysis of prognostic factors in patients with locally advanced cervical squamous cell carcinoma and development of a nomogram prediction model

**DOI:** 10.3389/fonc.2026.1833639

**Published:** 2026-09-10

**Authors:** Tingting Zou, Xupeng Wang, Haixia Wu, Zhuoran Li, Limin Chai, Zhiying Hao

**Affiliations:** 1 School of Pharmacy, Shanxi Medical University, Taiyuan, China; 2 Shanxi Medical University, Taiyuan, China; 3 Department of Pharmacy, Shanxi Province Cancer Hospital/Shanxi Hospital Affiliated to Cancer Hospital, Chinese Academy of Medical Sciences/Cancer Hospital Affiliated to Shanxi Medical University, Taiyuan, China

**Keywords:** LACSC, nomogram, overall survival, predictive model, prognostic factors

## Abstract

**Objective:**

To develop and validate a predictive model integrating patient demographics and clinical data for estimating 3-, 4-, and 5-year overall survival in patients with locally advanced cervical squamous cell carcinoma (LACSC).

**Methods:**

Clinical data from 670 LACSC patients at Shanxi Cancer Hospital were collected and then randomly assigned to a training cohort and an internal validation cohort at a 6:4 ratio by stratified sampling. Independent prognostic factors were identified using LASSO regression and multivariate Cox regression analysis, with which a predictive model was constructed. Model performance was assessed using the concordance index (C-index), receiver operating characteristic (ROC) curves, and calibration curves. Clinical utility was evaluated via decision curve analysis (DCA).

**Results:**

A robust prognostic model was developed and visualized as a nomogram comprising six variables: NEUT, MONO, CA125, SII, lymph node metastasis status, and treatment modality. Patients were stratified into high- and low-risk groups based on the median risk score in the training cohort. The high-risk group exhibited significantly poorer overall survival (OS) in both cohorts (*P* < 0.05).

**Conclusion:**

A clinical predictive model was established to estimate 3-, 4-, and 5-year survival rates for LACSC patients.

## 1 Introduction

Cervical cancer is the fourth most common cancer among women worldwide, posing a major threat to women’s health (1). A major breakthrough in past research was the identification of persistent HPV infection as the etiological cause of the disease. A synthesis of virological, molecular, and clinical epidemiological studies provides clear evidence that cervical cancer arises from long-term, unresolved infection with specific HPV genotypes (2). Histologically, cervical cancer is classified into squamous cell carcinoma, adenocarcinoma, adenosquamous carcinoma, and other rare types, with squamous cell carcinoma accounting for approximately 80%. Most patients are diagnosed at locally advanced stages, which are associated with poor prognosis (3, 4). Concurrent chemoradiotherapy (CCRT) with platinum-based chemotherapy is the standard treatment regimen for LACSC patients, but the risks of local recurrence and distant metastasis remain high (5). Currently, treatment options for LACSC mainly include CCRT, surgery, and targeted immunotherapy. To improve treatment outcomes and quality of life, there is an urgent clinical need for a prognostic model capable of predicting survival, which would help physicians assess the risk of disease progression, treatment response, and survival expectations, thereby facilitating personalized treatment planning. This study aims to develop a survival model for LACSC patients receiving curative-intent treatment, which can be used to evaluate prognosis and inform clinical decision-making.

## 2 Methods

### 2.1 Patient selection

This study included 670 patients diagnosed with LACSC at Shanxi Cancer Hospital between January and December 2020. Inclusion criteria were: (1) age ≥ 18 years; (2) newly diagnosed LACSC with FIGO stage (2018) IB_3_ to IVA; (3) received initial treatment with curative intent; (4) available clinical characteristics and follow-up data. Exclusion criteria were: (1) other pathological types; (2) prior or concurrent malignancies; (3) treatment interruption or abandonment; (4) missing clinical data; (5) refusal of follow-up. The primary endpoint was OS, defined as the time from diagnosis date to death from any cause or the date of last follow-up. The 670 patients who met the inclusion criteria were randomly assigned to a training cohort and an internal validation cohort at a 6:4 ratio via stratified random sampling. Follow-up was conducted by telephone, and the last follow-up date was December 31, 2025. This retrospective study was approved by the Ethics Committee of Shanxi Cancer Hospital, and the requirement for informed consent was waived (Ethics approval number: KY2026006).

### 2.2 Clinical parameter collection

Pretreatment baseline clinical data were collected, encompassing demographic and lifestyle characteristics (age, weight, height, BMI, smoking history, alcohol consumption history, and family history), reproductive and comorbidity status (menopausal status and comorbidities), and tumor-related variables (FIGO stage, maximum tumor diameter, tumor type, HPV infection status, lymph node metastasis status, and treatment regimen). Laboratory parameters comprised albumin (ALB), lactate dehydrogenase (LDH), hemoglobin (Hb), platelet count (PLT), absolute neutrophil count (NEUT), absolute lymphocyte count (LYMPH), absolute monocyte count (MONO), fibrinogen (FIB-C), D-dimer, squamous cell carcinoma antigen (SCC-Ag), carcinoembryonic antigen (CEA), carbohydrate antigen 19-9 (CA19-9), carbohydrate antigen 125 (CA125), and cytokeratin 19 fragment (CYFRA21-1). In addition, the following derived indices were calculated: neutrophil-to-lymphocyte ratio (NLR), platelet-to-lymphocyte ratio (PLR), lymphocyte-to-monocyte ratio (LMR), albumin-to-fibrinogen ratio (AFR), prognostic nutritional index (PNI), systemic immune-inflammation index (SII), and systemic inflammatory response index (SIRI). The normal reference ranges and calculation formulas for all these variables are presented in **Table 1**.

**Table 1:** Normal reference ranges and the corresponding calculation formulas.

Variables	Normal reference ranges or the corresponding calculation formulas
ALB	40.0-55.0 g/L
LDH	120.0-250.0 U/L
HB	115–150 g/L
PLT	125-350×10^9/L
NEUT	1.80-6.30×10^9/L
LYMPH	1.10-3.20×10^9/L
MONO	0.10-0.60×10^9/L
FIB-C	2.00-4.00 g/L
D-Dimer	0–550 ng/mL
SCC-Ag	0-2.70 ng/mL
CEA	0-3.00 ug/L
CA19-9	0-30.00 U/mL
CA125	0-35.00 U/mL
CYFRA21-1	0-3.30 ng/mL
NLR	NEUT/LYMPH
PLR	PLT/LYMPH
LMR	LYMPH/MONO
AFR	ALB/FIB-C
PNI	ALB+5×LYMPH
SII	(PLT×NEUT)/LYMPH
SIRI	(NEUT×MONO)/LYMPH

### 2.3 Data analysis

**Figure 1:**
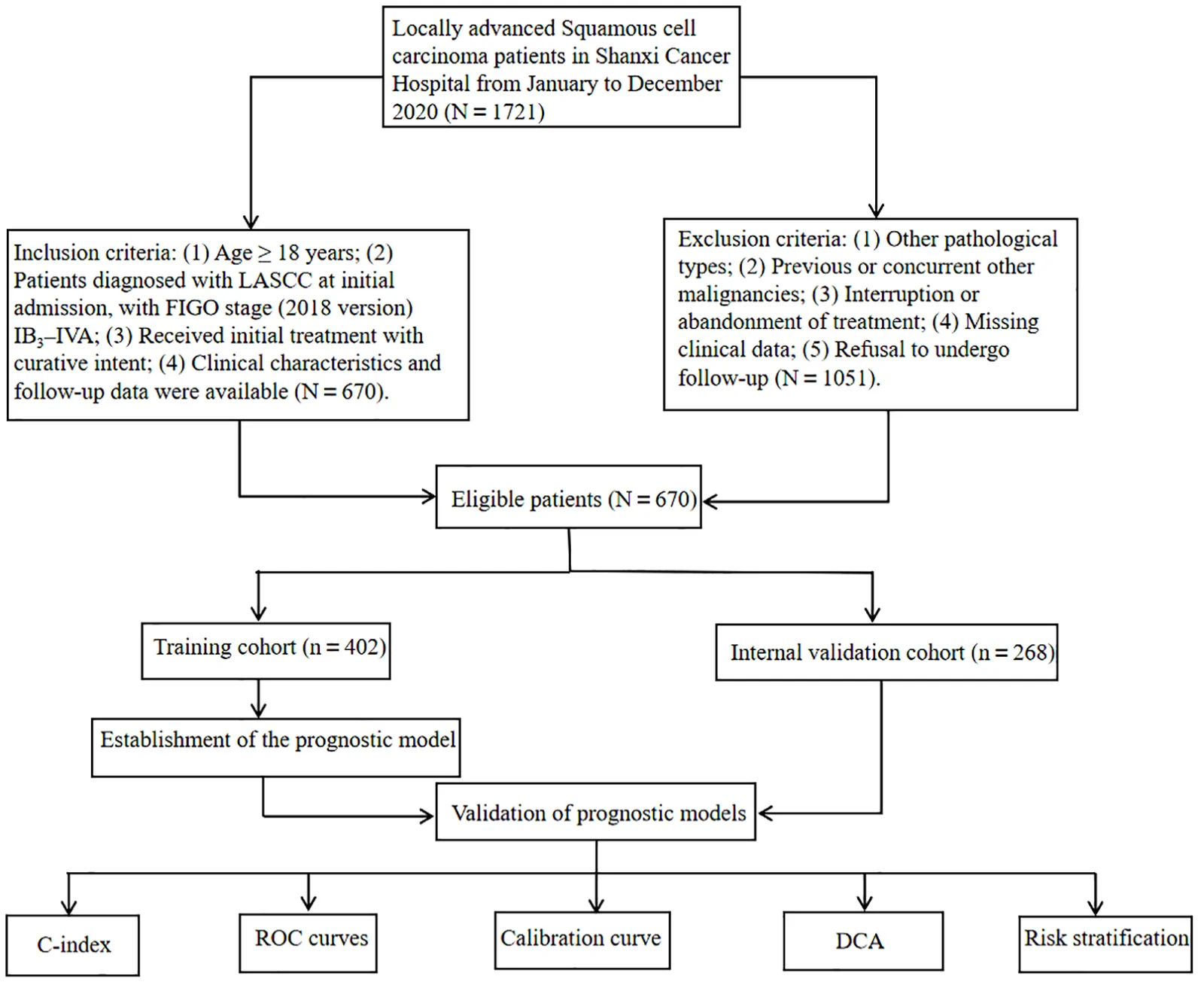
Flowchart of participant inclusion and exclusion.

Statistical analyses were performed using R software (version 4.4.3). Continuous variables were expressed as mean ± standard deviation, and categorical variables as counts (n) or percentages (%). Differences in patient characteristics between the training and internal validation cohorts were compared using the t-test (for continuous variables) and the chi-square test (for categorical variables). LASSO regression and multivariate Cox regression analyses were used to identify clinicopathological features significantly associated with survival. The selected variables were incorporated into a nomogram to predict 3-, 4-, and 5-year OS rates for LACSC patients. Model discrimination was assessed using the C-index and the area under the receiver operating characteristic curve (AUC); calibration curves were plotted to evaluate the agreement between nomogram-predicted probabilities and actual observed outcomes; and DCA was performed to evaluate the clinical net benefit. A risk score was calculated for each patient based on the nomogram, and patients were stratified into risk groups using the median risk score from the training cohort as the cutoff. Kaplan-Meier survival analysis was used to determine whether OS differed significantly between risk groups. The flowchart of patient selection and study design is shown in **Figure 1**.

## 3 Result

### 3.1 Participant characteristics

A total of 678 patients with LACSC were initially enrolled, of whom 8 were lost to follow-up (loss-to-follow-up rate: 1.18%), resulting in 670 patients finally included for statistical analysis. These patients were divided into a training set (n = 402) and an internal validation set (n = 268) at a 6:4 ratio using stratified random sampling. No significant differences in demographic or clinicopathological characteristics were observed between the two cohorts (*P* > 0.05), as shown in **Table 2**.

**Table 2:** Patient baseline data characteristics.

Clinical characteristics	Training set (n = 402)	Internal validation set (n = 268)	*P* value
Age	54.43 ± 11.39	54.82 ± 10.17	0.647
Weight	60.57 ± 9.41	61.02 ± 9.69	0.547
Height	156.84 ± 5.18	157.30 ± 5.12	0.261
BMI	24.61 ± 3.53	24.64 ± 3.58	0.902
Smoking History			0.144
Yes	17 (4.2%)	5 (1.9%)	
No	385 (95.8%)	263 (98.1%)	
Alcohol History			0.665
Yes	2 (0.5%)	0 (0.0%)	
No	400 (99.5%)	268 (100.0%)	
Family History			0.726
Yes	9 (2.2%)	8 (3.0%)	
No	393 (97.8%)	260 (97.0%)	
Menopause			0.961
Yes	254 (63.2%)	168 (62.7%)	
No	148 (36.8%)	100 (37.3%)	
Comorbidities			0.161
Yes	151 (37.6%)	116 (43.3%)	
No	251 (62.4%)	152 (56.7%)	
FIGO Stage			0.218
I B3	32 (8.0%)	13 (4.9%)	
II A1	83 (20.6%)	50 (18.7%)	
II A2	43 (10.7%)	41 (15.3%)	
II B	36 (9.0%)	23 (8.6%)	
III A	3 (0.7%)	3 (1.1%)	
III B	43 (10.7%)	20 (7.5%)	
III C1p	93 (23.1%)	68 (25.4%)	
III C1r	46 (11.4%)	41 (15.3%)	
III C2p	4 (1.0%)	3 (1.1%)	
III C2r	16 (4.0%)	6 (2.2%)	
IV A	3 (0.7%)	0 (0.0%)	
Maximum Tumor Diameter	4.39 ± 1.43	4.37 ± 1.39	0.878
Tumor Subtype			0.274
Nodular Type	99 (24.6%)	53 (19.8%)	
Endophytic Type	232 (57.7%)	168 (62.7%)	
Exophytic	43 (10.7%)	23 (8.6%)	
Other	28 (7.0%)	24 (9.0%)	
HPV			0.094
Positive	372 (92.5%)	237 (88.4%)	
Negative	30 (7.5%)	31 (11.6%)	
ALB			0.471
Normal	315 (78.4%)	217 (81.0%)	
Abnormal	87 (21.6%)	51 (19.0%)	
LDH			0.817
Normal	389 (96.8%)	261 (97.4%)	
Abnormal	13 (3.2%)	7 (2.6%)	
HB			0.232
Normal	277 (68.9%)	197 (73.5%)	
Abnormal	125 (31.1%)	71 (26.5%)	
NEUT			0.878
Normal	339 (84.3%)	228 (85.1%)	
Abnormal	63 (15.7%)	40 (14.9%)	
PLT			0.791
Normal	330 (82.1%)	217 (81.0%)	
Abnormal	72 (17.9%)	51 (19.0%)	
LYMPH			0.867
Normal	351 (87.3%)	236 (88.1%)	
Abnormal	51 (12.7%)	32 (11.9%)	
MONO			0.855
Normal	360 (89.6%)	242 (90.3%)	
Abnormal	42 (10.4%)	26 (9.7%)	
FIB-C			0.399
Normal	342 (85.1%)	235 (87.7%)	
Abnormal	60 (14.9%)	33 (12.3%)	
D-Dimer			0.087
Normal	375 (93.3%)	259 (96.6%)	
Abnormal	27 (6.7%)	9 (3.4%)	
PNI	51.20 ± 5.27	51.85 ± 5.49	0.122
NLR	2.69 ± 1.76	2.55 ± 1.61	0.313
PLR	166.70 ± 79.83	164.28 ± 74.26	0.692
LMR	5.42 ± 2.80	5.36 ± 2.32	0.774
SII	779.87 ± 779.46	741.84 ± 599.61	0.499
SIRI	1.16 ± 1.37	1.08 ± 0.98	0.408
AFR	13.66 ± 3.47	13.89 ± 3.35	0.391
SCC-Ag			0.085
Normal	207 (51.5%)	119 (44.4%)	
Abnormal	195 (48.5%)	149 (55.6%)	
CEA			0.552
Normal	272 (67.7%)	188 (70.1%)	
Abnormal	130 (32.3%)	80 (29.9%)	
CA19-9			0.417
Normal	359 (89.3%)	233 (86.9%)	
Abnormal	43 (10.7%)	35 (13.1%)	
CA125			0.831
Normal	372 (92.5%)	250 (93.3%)	
Abnormal	30 (7.5%)	18 (6.7%)	
CYFRA21-1			0.396
Normal	98 (24.4%)	74 (27.6%)	
Abnormal	304 (75.6%)	194 (72.4%)	
Lymph node metastasis status			0.266
Pelvic lymph node metastasis	139 (34.6%)	109 (40.7%)	
Para-aortic lymph node metastasis	9 (2.2%)	3 (1.1%)	
Pelvic and para-aortic lymph node metastasis	14 (3.5%)	6 (2.2%)	
No metastasis	240 (59.7%)	150 (56.0%)	
Treatment modality			0.417
Concurrent chemoradiotherapy	125 (31.1%)	71 (26.5%)	
Curative surgery	41 (10.2%)	40 (14.9%)	
Curative surgery plus adjuvant therapy	139 (34.6%)	95 (35.4%)	
Neoadjuvant therapy plus surgery	30 (7.5%)	16 (6.0%)	
Neoadjuvant therapy plus surgery and adjuvant therapy	31 (7.7%)	19 (7.1%)	
Chemotherapy alone or radiotherapy alone	36 (9.0%)	27 (10.1%)	

Among the 670 patients enrolled from Shanxi Cancer Hospital, the majority were middle-aged and elderly, with those aged 50–86 years accounting for 70.29%. Regarding FIGO stage, most patients were stage IIIC (41.34%) and stage IIA (32.38%). HPV testing was positive in 90.89% of patients. Among patients with lymph node metastasis, 88.57% had pelvic lymph node metastasis (PLNM). In terms of treatment modality, 34.9% of patients received radical surgery plus adjuvant therapy, while 29.2% underwent CCRT.

### 3.2 Independent prognostic factors for screening model construction

**Figure 2:**
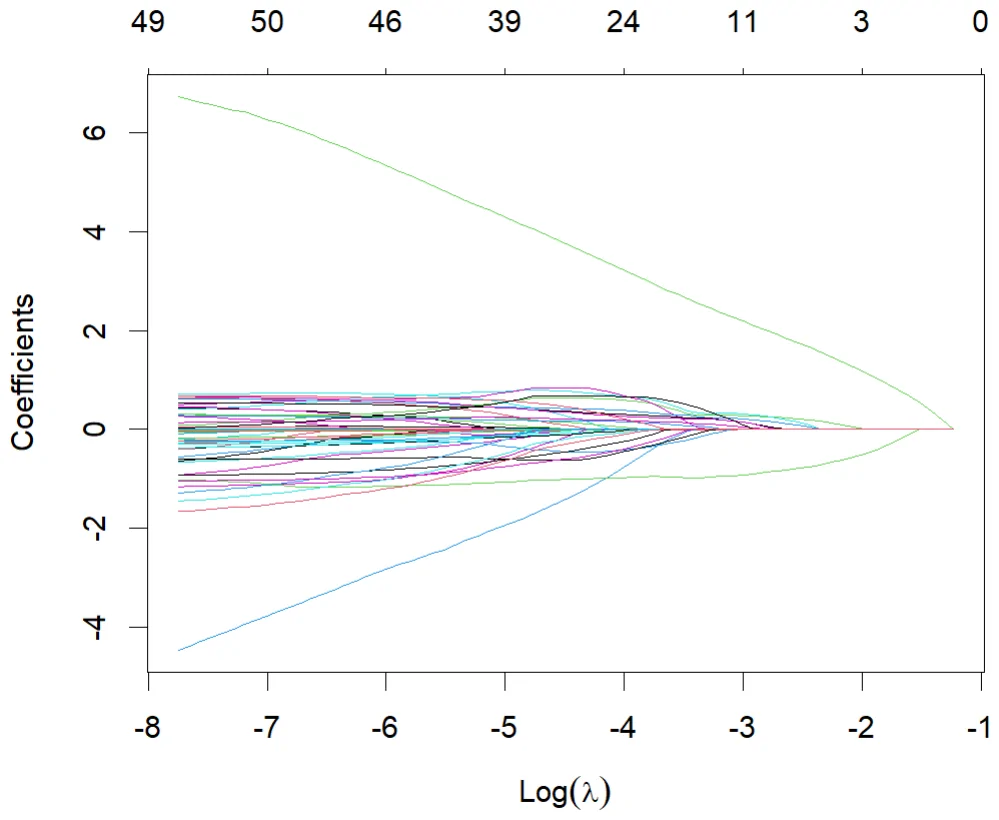
Dynamic process diagram of variable selection in lasso regression.

**Figure 3:**
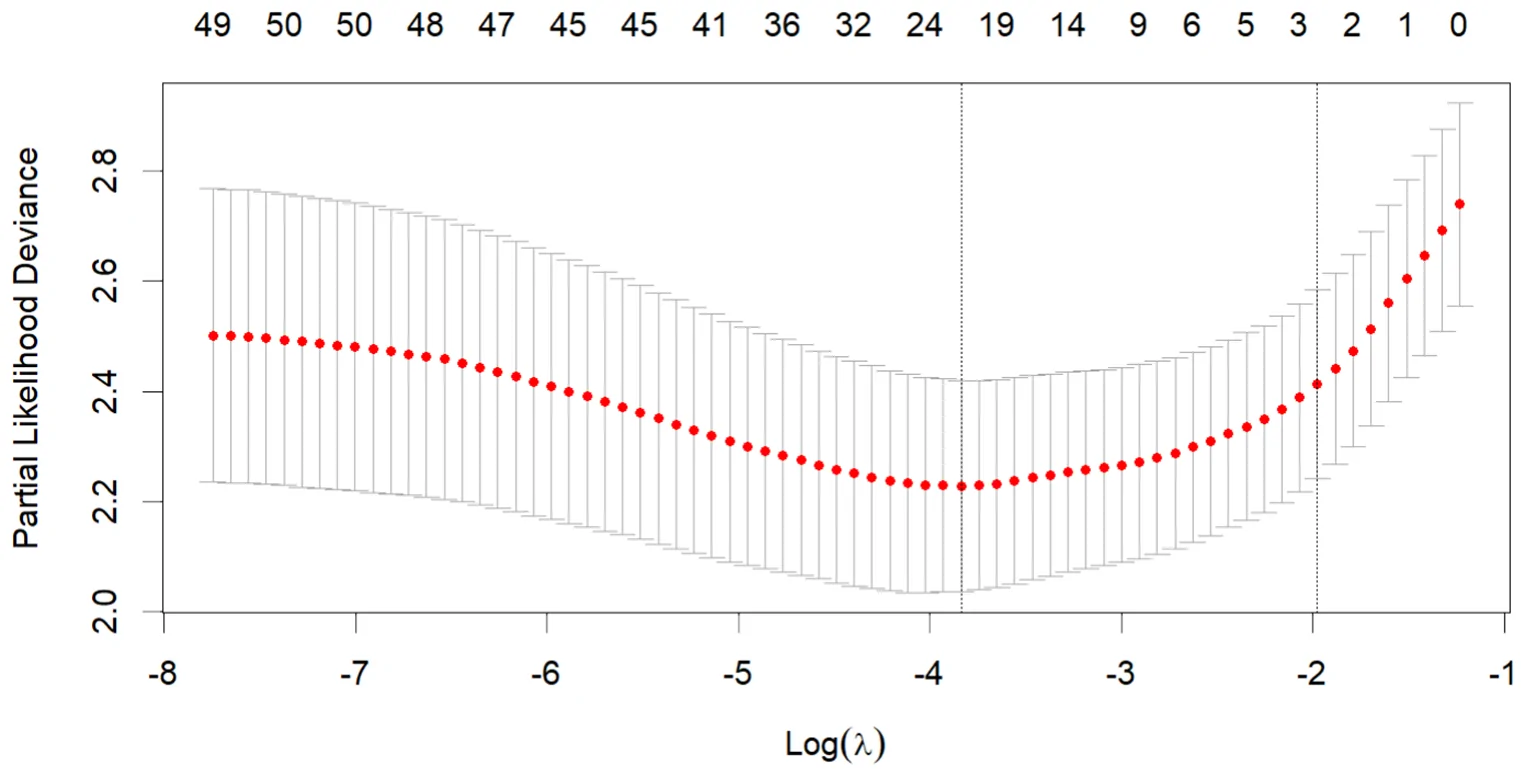
Diagram of the selection process for the optimal cross-validation parameter λ.

LASSO regression was used to screen clinicopathological features significantly associated with patient survival in the training cohort. The coefficient paths for each variable are shown in **Figure 2**. The iterative analysis employed 10-fold cross-validation, and the λ value of 0.02154646 (logλ = -3.83) was selected as it yielded the model with the most retained variables and the smallest binomial deviance (**Figure 3**). Ultimately, 19 variables were selected via LASSO regression, including NEUT, lymph node metastasis status, and treatment modality. These 19 variables were then subjected to multivariate Cox regression analysis, and variables with *P* < 0.05 were considered significantly associated with patient survival and were incorporated into the final model. The results revealed that NEUT, MONO, CA125, SII, lymph node metastasis status, and treatment modality were independent prognostic factors for LACSC patients, as shown in **Table 3**.

**Table 3:** Cox regression multivariate analysis.

Clinical characteristics	HR (95% CI)	*P* value
Weight	0.980 (0.959, 1.001)	0.066
FIGO stage		
IIIC1r	1.934 (0.820, 4.564)	0.132
IIIC2p	0.000 (0.000, Inf)	0.995
IIIC2r	3.141 (0.868, 11.364)	0.081
IVA	2.932 (0.482, 17.836)	0.243
Tumor subtype		
Exophytic	0.517 (0.218, 1.224)	0.134
Other	1.469 (0.810, 2.667)	0.206
ALB	1.432 (0.906, 2.262)	0.124
CEA	1.258 (0.822, 1.924)	0.290
NEUT	2.246 (1.259, 4.005)	0.006
MONO	1.828 (1.180, 2.832)	0.015
CA125	1.868 (1.017, 3.432)	0.044
SII	1.521 (1.167, 1.981)	0.015
Lymph node metastasis status		
Para-aortic lymph node metastasis	1.997 (0.562, 7.090)	0.285
No metastasis	0.192 (0.102, 0.360)	< 0.001
Treatment modality		
Concurrent chemoradiotherapy	0.379 (0.203, 0.707)	0.002
Curative surgery plus adjuvant therapy	0.434 (0.212, 0.888)	0.022
Neoadjuvant therapy plus surgery	1.276 (0.548, 2.974)	0.572
Neoadjuvant therapy plus surgery and adjuvant therapy	0.423 (0.155, 1.150)	0.092

### 3.3 Model creation and validation

**Figure 4:**
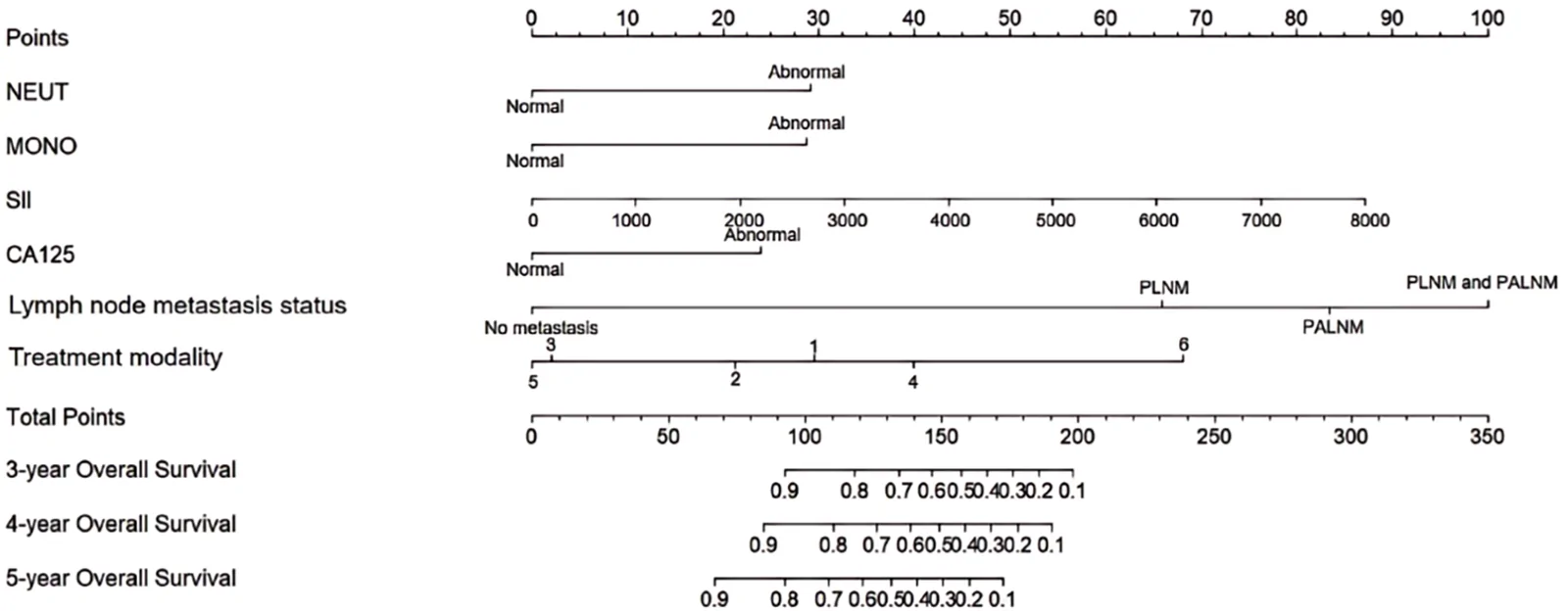
Nomogram (treatment modality: 1-concurrent chemoradiotherapy; 2-curative surgery; 3-curative surgery plus adjuvant therapy; 4-neoadjuvant therapy plus surgery; 5-neoadjuvant therapy plus surgery and adjuvant therapy; 6-chemotherapy alone or radiotherapy alone).

The nomogram for predicting survival in LACSC patients was constructed based on the above six variables, as shown in **Figure 4**. By summing the points assigned to each of the six variables, the 3-, 4-, and 5-year OS rates can be estimated.

The performance of the model was further validated using the C-index, time-dependent ROC curves, and calibration curves. The C-index values for the training and internal validation cohorts were 0.794 (95% CI: 0.751-0.838) and 0.781 (95% CI: 0.729-0.833), respectively, indicating good discriminative ability, substantially exceeding random chance. The results were stable and reliable, with no evidence of significant bias, and demonstrated strong generalizability in cross-validation.

**Figure 5:**
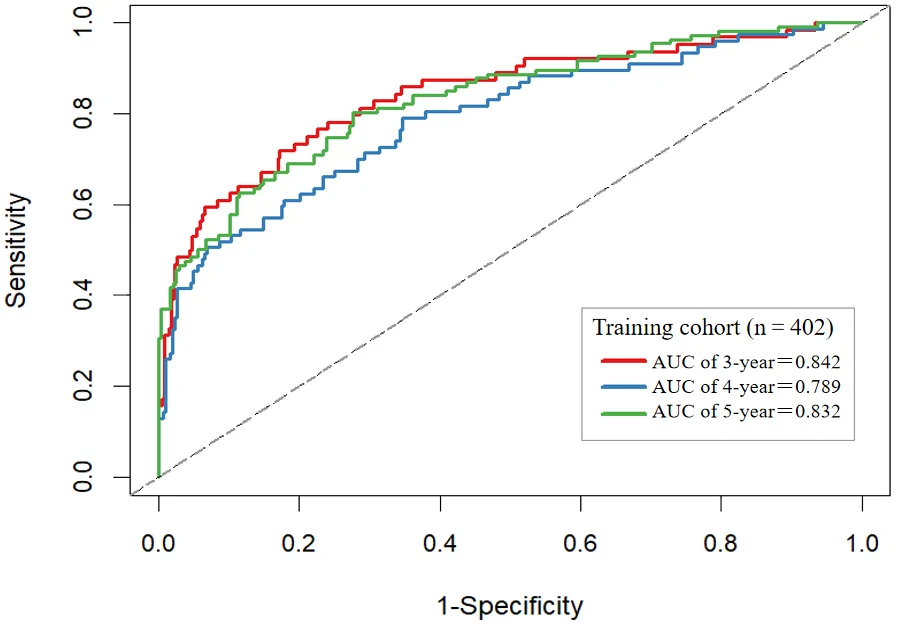
The time-dependent ROC curves of the nomogram predicting OS at 3-year and 4-year and 5-year in the training cohort.

**Figure 6:**
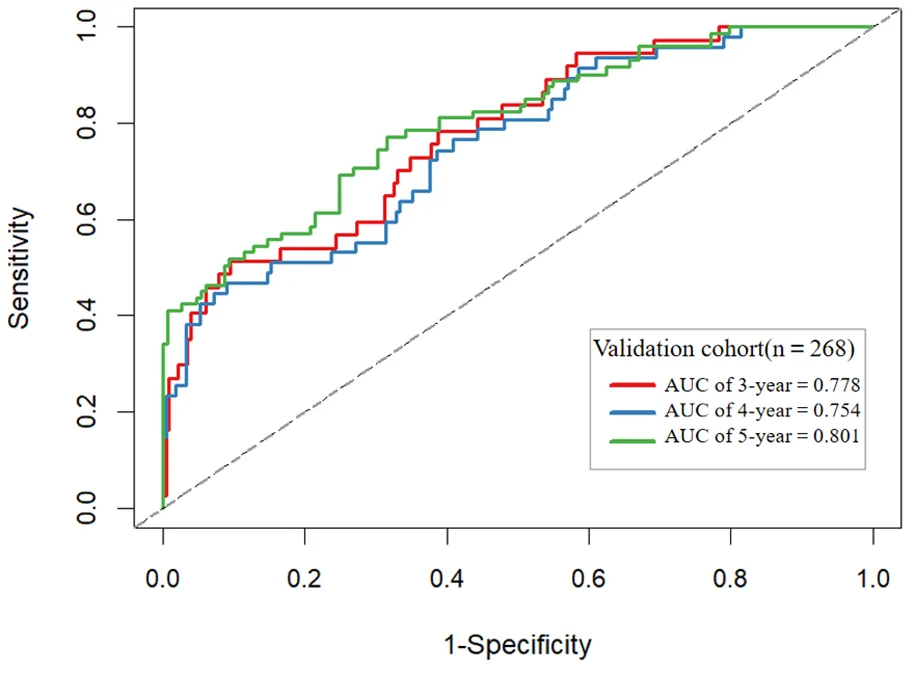
The time-dependent ROC curves of the nomogram predicting OS at 3-year and 4-year and 5-year in the internal validation cohort.

**Figures 5** and **6** show the time-dependent ROC AUCs of the nomogram for predicting 3-, 4-, and 5-year OS rates in both cohorts. In the training cohort, the AUCs were 0.842 (95% CI: 0.782-0.901) for 3-year OS, 0.789 (95% CI: 0.728-0.850) for 4-year OS, and 0.832 (95% CI: 0.783-0.880) for 5-year OS. In the internal validation cohort, the corresponding AUCs were 0.778 (95% CI: 0.698-0.857), 0.754 (95% CI: 0.678-0.831), and 0.801 (95% CI: 0.739-0.863). These C-index values and AUC values indicate that the prognostic model possesses favorable discriminative ability for survival in LACSC patients.

**Figure 7:**
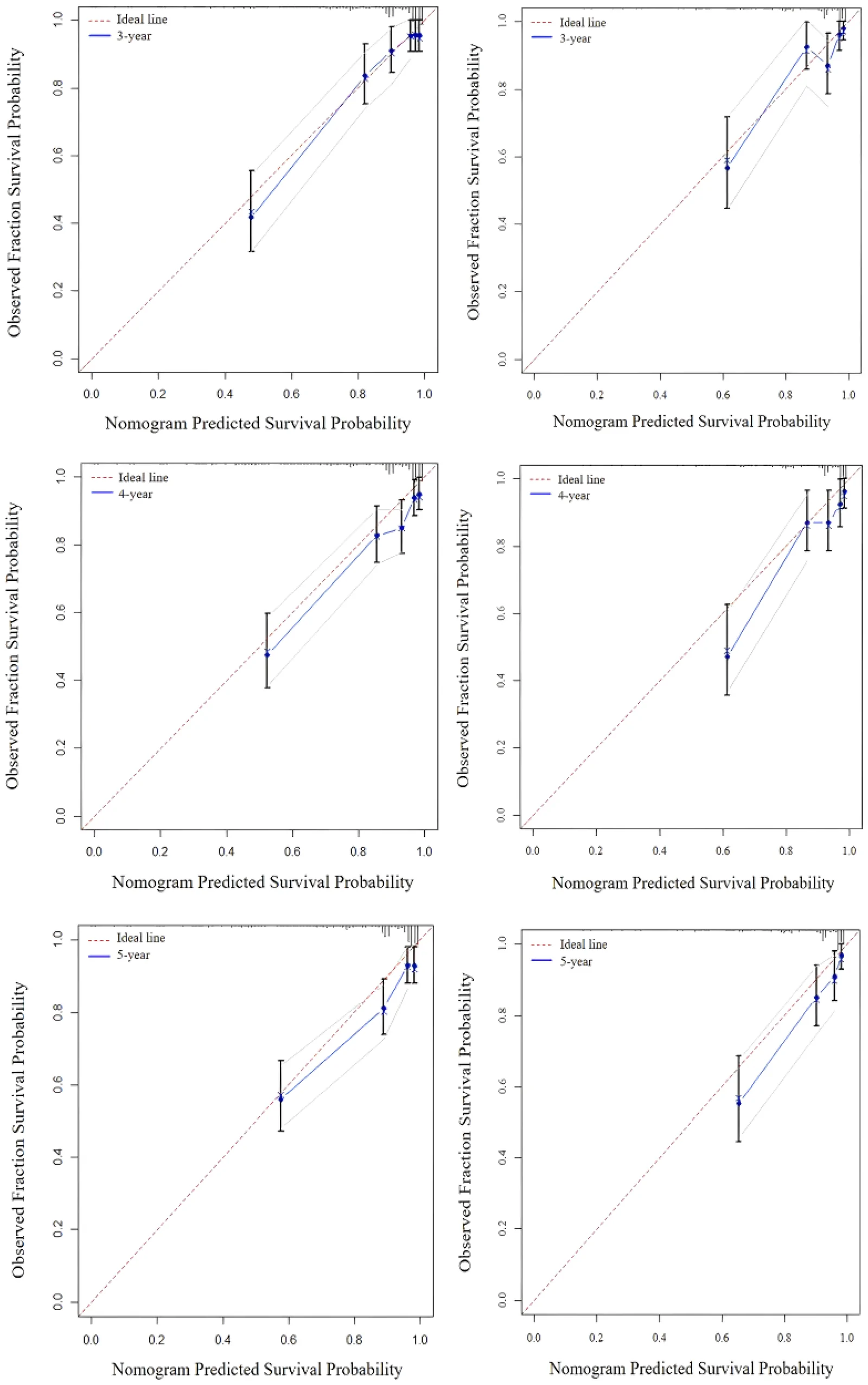
The calibration curves for predicting OS at (left) 3-year and 4-year and 5-year in the training cohort, and at (right) 3-year 4-year and 5- year in the internal validation cohort.

**Figure 7** displays the calibration curves of the predictive model, comparing the actual OS rates with the predicted probabilities at 3, 4, and 5 years in both cohorts. The results demonstrated good agreement between the nomogram-predicted survival rates and the observed survival rates.

**Figure 8:**
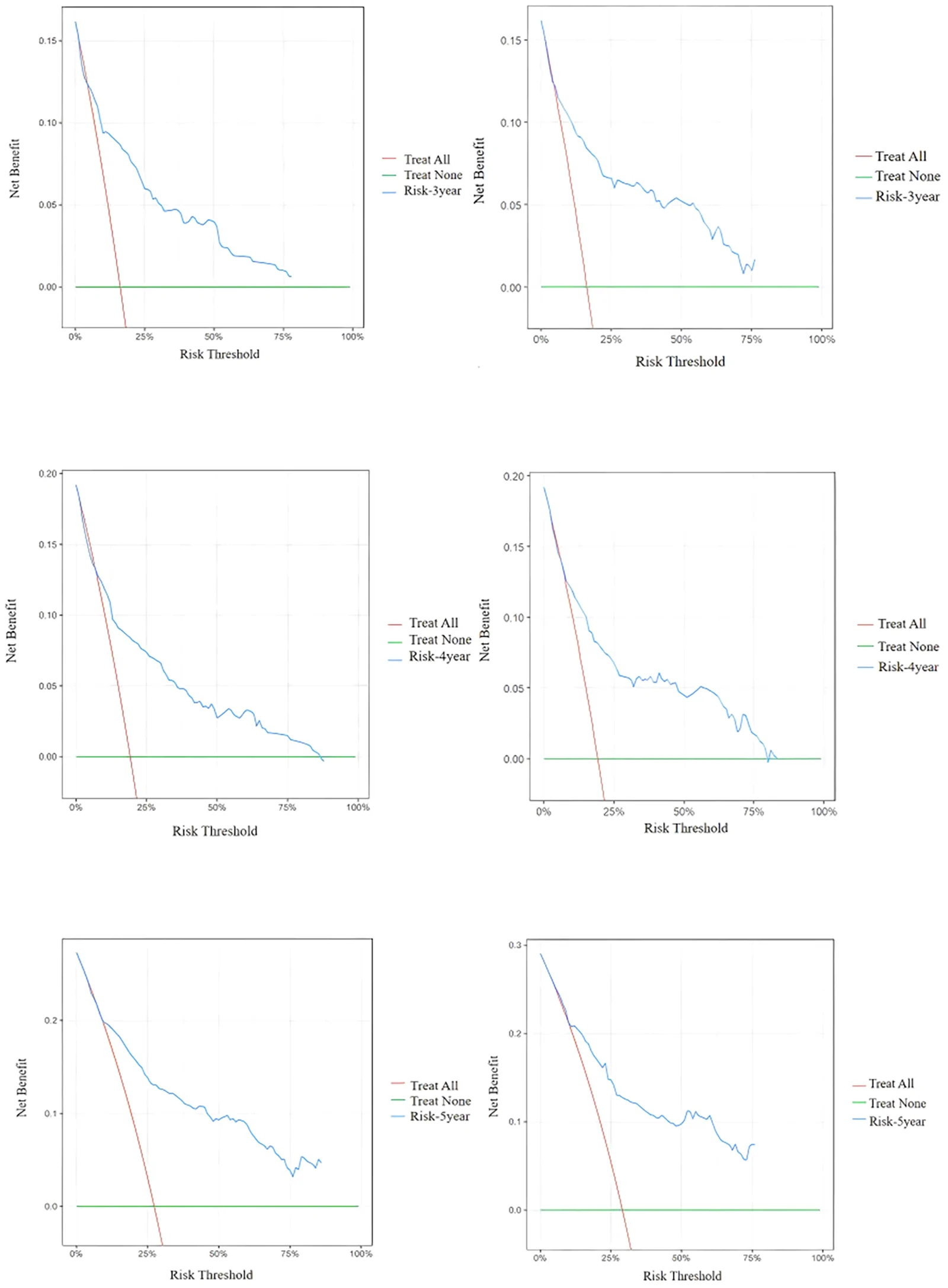
DCA The training cohort (left) and internal validation cohort (right).

DCA was applied to evaluate the clinical utility of the predictive model. The horizontal line represents the assumption that all patients are negative (i.e., no intervention), with a net benefit of zero; the diagonal line represents the assumption that all patients are positive (i.e., intervention for all), with a net benefit corresponding to a downward-sloping line. The farther the decision curve deviates from these two extreme lines, the greater the clinical application value of the nomogram. As shown in **Figure 8**, the constructed model improved the predictive ability for LACSC patient survival within a range of risk threshold probabilities.

### 3.4 Risk stratification based on nomogram

**Figure 9:**
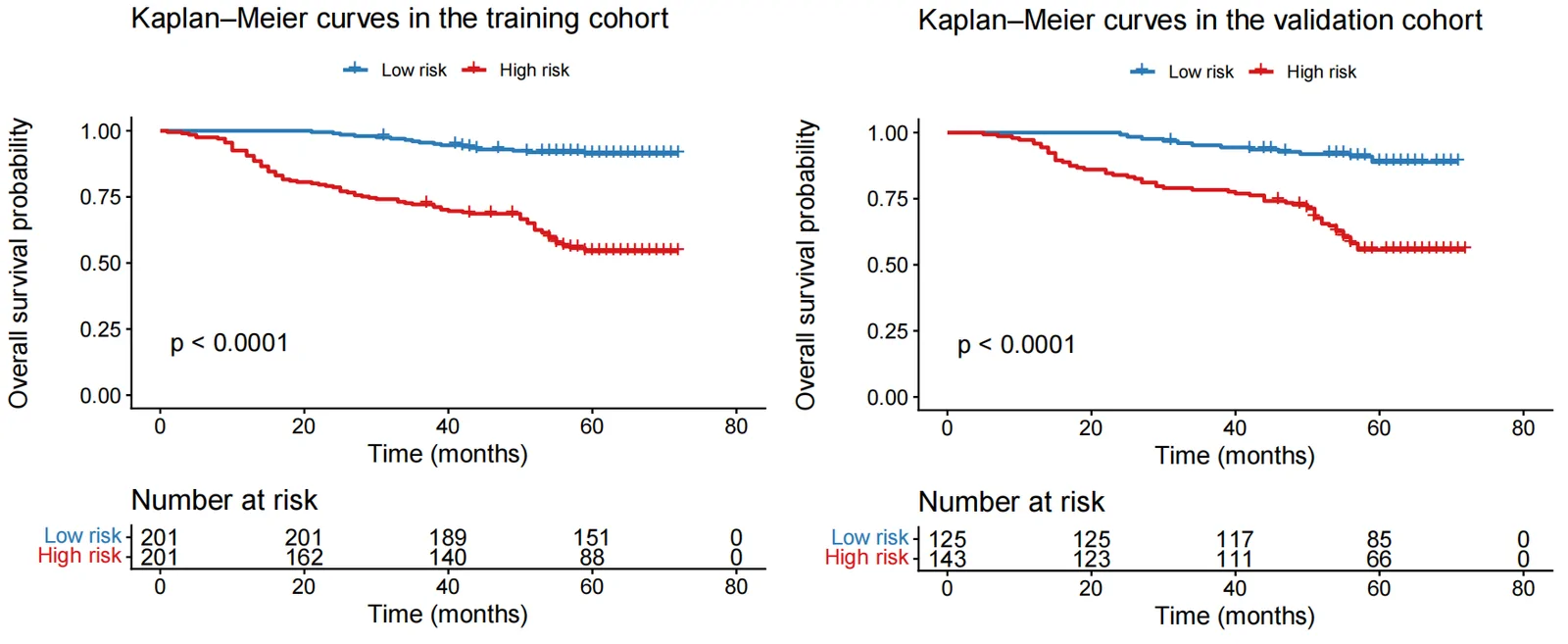
Kaplan-Meier curves for correlation with OS for the low and high-risk groups in the training cohort (left), internal validation cohort (right).

Risk scores were calculated for all patients based on the nomogram. With the median OS risk score from the training cohort serving as the cutoff value, patients were stratified into high-risk and low-risk groups. Kaplan-Meier survival analysis (**Figure 9**) revealed significant differences in OS between the two risk groups, indicating that the nomogram facilitates accurate risk stratification for LACSC patients.

## 4 Discussion

This study aimed to develop an individualized predictive model to better estimate survival outcomes for LACSC patients. LACSC patients generally have poor prognosis and frequently present with lymph node metastasis, most commonly involving pelvic and para-aortic regions. Variables were selected using LASSO regression and multivariate Cox regression analysis, and the final model incorporated six variables: NEUT, MONO, CA125, SII, lymph node metastasis status, and treatment modality.

In this study, laboratory findings in LACSC patients revealed that normal levels of NEUT and MONO were protective factors for survival prognosis, whereas abnormalities indicated poorer outcomes. It is increasingly evident that NEUT and other myeloid cell populations play important roles in cancer progression and treatment response. Several studies have reported an association between elevated pretreatment circulating neutrophil levels and poor prognosis following radiotherapy or chemotherapy in various tumor types (6, 7). In cervical cancer, extremely high pretreatment neutrophil levels have been linked to lower survival rates after adjusting for other prognostic factors (8). Additionally, studies have shown that elevated monocyte count is an independent prognostic factor for overall survival in patients with stage IB and IIA cervical cancer (9), and higher pretreatment circulating monocyte count is independently associated with poor prognosis in LACSC patients (10).

In recent years, there has been growing research on the tumor microenvironment (TME). The TME is a highly heterogeneous and complex ecosystem composed of innate immune cells (e.g., neutrophils, macrophages, dendritic cells (DCs), myeloid-derived suppressor cells (MDSCs), and natural killer (NK) cells), adaptive immune cells (comprising T and B cells), stromal cells, and the extracellular matrix (ECM) (11, 12). Neutrophils originate from granulocyte-monocyte progenitors (GMPs) derived from hematopoietic stem cells and undergo successive stages—myeloblast, promyelocyte, myelocyte, metamyelocyte, and band cell—under the regulation of granulocyte colony-stimulating factor (G-CSF), ultimately maturing into polymorphonuclear neutrophils. Recruited by various cytokines and chemokines secreted by tumors, neutrophils infiltrate the TME and become tumor-associated neutrophils (TANs) (13). Similar to the M1/M2 polarization of macrophages, neutrophils also exhibit two functional states, N1 and N2, with N1 possessing antitumor activity and N2 exerting protumor effects. N1 neutrophils can kill cancer cells by releasing reactive oxygen species (ROS) and neutrophil extracellular traps (NETs), and they enhance antitumor immune responses by secreting CXCL9/10 to recruit CD8-positive T cells (14, 15).

The TME recruits monocytes to tumor sites via inflammatory signals and induces their further differentiation into tumor-associated macrophages (TAMs) or dendritic cells. Tumor cells can also attract monocytes to the TME through extracellular vesicles and can induce their differentiation into TAMs with a protumor phenotype. Monocytes regulate immune responses in the TME through multiple mechanisms: both classical and non-classical monocytes can secrete immunosuppressive cytokines, while immature monocytes (monocytic MDSCs) expand peripherally and accumulate within tumors, leading to local immunosuppression; monocytes can directly “hijack” certain components of cancer cells (including antigens) and present them to T cells, thereby enhancing antitumor immunity; and tumor-infiltrating monocytes differentiate into TAMs within the TME, which exhibit functional plasticity along a dynamic continuum between pro-inflammatory (M1-like) and immunosuppressive (M2-like) states, influenced by tumor signals and the spatial microenvironment (16). TANs and TAMs have become important avenues for next-generation immunotherapy.

CA125 is a common biomarker used in the detection of tumors of the female reproductive system, and its serum levels are significantly elevated in patients with cervical cancer (17, 18). Therefore, CA125 is widely used as a tumor marker for cervical cancer in routine clinical practice.

In recent years, the systemic immune-inflammation index (SII), derived from peripheral lymphocyte, neutrophil, and platelet counts, has been recognized as a superior indicator of local immune response and systemic inflammation, as it has been validated in various tumor types, including hepatocellular carcinoma (19), esophageal cancer (20, 21), colorectal cancer (22), and small cell lung cancer (23). Neutrophils are a key inflammatory driver component of the SII, contributing to endothelial dysfunction and vascular wall degradation through the secretion of inflammatory mediators. Neutrophils also promote inflammation-related pathological processes via interactions with platelets, proteolytic cleavage of coagulation factors, release of prothrombotic molecules, and facilitation of monocyte infiltration (24). Platelets are an important component of the SII, reflecting thrombosis and vascular inflammation. They release chemokines, proinflammatory cytokines, and platelet-derived growth factors, which contribute to endothelial cell exhaustion. Platelet activation and aggregation not only participate in thrombus formation but also promote local and systemic inflammatory responses through the release of various inflammatory mediators (25). Lymphocytes are a key component of the SII, reflecting the host’s immune surveillance capacity and antitumor immunity. Studies have shown that SII is closely associated with alterations in CD8-positive T lymphocyte subsets. Patients with shorter overall survival exhibit a significant reduction in the percentage of CD8-positive T lymphocytes and their effector subsets, accompanied by an increase in naive subsets. An elevated SII after chemotherapy is closely correlated with a decreased CD4/CD8 ratio, and both can serve as potential immunoinflammatory indicators for prognosis assessment (26). In prognostic studies of cervical cancer patients, evidence has demonstrated that SII can be used to evaluate the prognosis of patients after radical surgery, and SII has been shown to be more effective and accurate in predicting patient outcomes (27).

The 2018 International Federation of Gynecology and Obstetrics (FIGO) staging system for cervical cancer stipulates that patients with lymph node metastasis should be assigned to stage IIIC or higher (28). Due to the unique anatomical structure of the pelvis, cancer cells primarily spread via lymphatic channels to the pelvic and para-aortic lymph nodes (29). Previous studies have shown that cervical cancer patients with lymph node metastasis have higher recurrence rates and lower 5-year overall survival (30, 31). Therefore, lymph node metastasis status plays a critical role in the prognosis of cervical cancer patients.

In recent years, numerous predictive models have been developed for LACSC. One study constructed a nomogram based on radiomics from 18F-FDG PET, extracting radiomic features from pretreatment 18F-FDG PET images to calculate the Rad-score, which was then combined with clinical factors to build a combined prediction model (32). Another study developed a nomogram using MRI-based radiomics, retrospectively analyzing 122 LACSC patients, extracting radiomic features from pretreatment pelvic MRI to establish a Rad-score, and integrating clinical factors to create a predictive model (33). Additionally, a combined model integrating clinical and radiomics features was built based on texture analysis of ADC maps, enrolling 219 LACSC patients, extracting 22 first-order texture features from ADC maps, and combining them with clinical variables (34).

The prognostic prediction model for LACSC constructed in this study demonstrates substantial clinical utility. First, it innovatively focuses on systemic inflammatory response by integrating indicators such as NEUT, MONO, and SII, which, compared with NLR or PLR alone, more comprehensively reflect the tumor-related immune-inflammatory status. Second, the model is simple, efficient, and cost-effective, as the six ultimately included predictors are all routine clinical and laboratory parameters requiring no additional expenses, which thereby greatly enhances clinical decision-making efficiency. Furthermore, the study is based on a large sample size of over 600 cases, which ensures model robustness and reliability. Most importantly, the model circumvents the bottlenecks of high technical thresholds and limited generalizability associated with radiomics, returning to a clinically oriented approach, and is thus more suitable for individualized risk assessment in primary care and resource-limited settings. We hope that more internal and external cases can be collected in the future to validate and further calibrate the model’s performance.

## 5 Conclusion

The data used in this study were derived from real-world clinical practice, which can truly reflect the status of LACSC patients and are of high reference value. However, given the retrospective single-center design, external validation was lacking; the generalizability of the model is unclear, and the study population was limited to patients treated at our institution, which may inevitably introduce selection bias. The generalizability of the model in community primary care or non-Asian populations remains to be verified. Nevertheless, internal validation results showed good calibration, and DCA indicated a net benefit within the threshold probability range, suggesting that despite biases, the model still has reliable clinical utility at our center. Future multicenter prospective studies are needed to further validate the model’s performance in broader populations. Notably, the patient population at our center has significant regional socioeconomic characteristics. Since genetic testing and some high-resolution imaging examinations have not been included in the universal health insurance coverage in this region and are associated with high out-of-pocket costs, such tests are not mandatory as pretreatment baseline screening tools in clinical practice; they are selectively performed only for subgroups with disease progression or clear targeted indications. Consequently, the missing data rate for these variables is high, and they were not included in this study.

In summary, we successfully constructed and validated a predictive model for survival prognosis in LACSC patients, which provides a robust basis for treatment decisions in this patient population.

## Data Availability

The original contributions presented in the study are included in the article/supplementary material. Further inquiries can be directed to the corresponding author/s.
